# The Present Status of the Surgery of the Vermiform Appendix

**Published:** 1892-09

**Authors:** John A. Wyeth

**Affiliations:** New York; Professor of Surgery at the New York Polyclinic, Visiting Surgeon to Mt. Sinai Hospital


### *SELECTED ARTICLE.*

### THE PRESENT STATUS OF THE SURGERY OF THE VERMIFORM APPENDIX.

BY JOHN A. WYETH, M. D.,

Professor of Surgery at the New York Polyclinic, Visiting Surgeon to Mt. Sinai Hospital.

The achievements of modern surgery are the just pride of our profession; they have won for it and us the admiration and gratitude of mankind. Even in this wonderful age of mechanical invention, it is safe to say that in its proper sphere, the alleviation of suffering and the preservation of life, surgery has not been surpassed in marvelous progress. Ether, chloroform and cocaine have already saved millions of beings from untimely death and are destined for all time to compound the sum of human health and happiness.

One need go no further than the subject of this paper for a demonstration of the rapid progress in surgery. The morbid anatomy of the appendix vermiformis has only recently been understood, and with this knowledge came at once the great improvement in the operative treatment of its lesions.

The name of Dr. Simon Baruch, of New York City, should, in my opinion, stand prominent in the list of those to whom we are indebted for this very important advance. It was from a case in his practice that on Feb. 8, 1888, Dr. Henry B. Sands reported to the New York Surgical Society "*An account of a case in which recovery took place after laparotomy had been performed for septic peritonitis due to a perforation of the vermiform appendix.*"*

A proper conception of what we once termed "perityphlitis," now properly named *appendicitis* and *peri-appendicitis*, will depend upon a careful consideration of the anatomy of the organ in question. Quoting from Quain is this description: "Coming off from the inner and back part of the cæcum at its lower end is a narrow, round and tapering portion of the intestine named the appendix cæci or *vermiform appendix*. The width of this process is usually about that of a large quill, or rather more, and its length varies from three to six inches; these dimensions differing much in different cases. Its general direction is upwards and inwards behind the cæcum and after describing a few slight turns it ends in a blunt point. It is retained in its position by a small fold of peritoneum which forms its mesentery. The cæcal extremity is hollow as far as its extremity, and its cavity communicates with that of the cæcum by a small orifice sometimes guarded by a valvular fold of mucous membrane."

Of course, the anatomical relations of this organ vary from this most frequent position, but in the large proportion of subjects the above relation prevails.

In this unfortunate position, it cannot escape many accidental lesions. Its lumen communicating with the great pouch, the largest part of the alimentary canal, into which is being poured during a good portion of the time semi-liquid ingested matter,

*N. Y. Medical Journal, Feb. 18, 1888, p. 197.

and various undigested particles under considerable pressure, no matter what position the subject assumes, it is no wonder that these contents find their way into this organ. Once in, the tendency is to remain; for this diminutive process has little peristaltic power to empty itself. Hence by pressure of solid particles or by fæcal enteroliths formed by partial evaporation of the liquid contents, ulcers are formed and perforation, more or less rapid and complete, may occur with escape of gas or other contents, or without perforation inflammation may be instituted, which spreads through the walls and fires up the peritoneum in immediate contact. Moreover, pressure of the overlying and usually filled cæcum directly upon the appendix must be an additional factor in the interference with its proper nutrition and the resulting breaking down.

Again when distended with gas, or even when empty and small, such is its unfortunate position that this pressure may shut off a proper blood supply and cause gangrene, general or localized. Pressure is undoubtedly increased in the sitting posture and this may, in part, account for the frequency of appendicitis in those of sedentary habits.

From the above we can satisfactorily explain all the various lesions which are found post mortem or during operative exploration, from the mild catarrhal inflammation of the lining membrane, to the deeper process speading without perforation to the peritoneum; the general rapid gangrene of the entire organ with rapidly developing peritonitis, rupture and collapse; the slow perforation by direct pressure of a foreign substance; and ulceration and perforation as a result of local gangrene in an appendix either empty or containing only a small quantity of gas.

*Catarrhal appendicitis* is the simplest and most innocent variety. The symptoms vary with the intensity of the catarrhal process and with its recurrence. Pain is usually mild, fever not present, and tumefaction or local abdominal resistance absent. When the inflammatory process involves the entire wall of the appendix and the peritoneum is commencing to be involved, pain becomes a more prominent symptom, tenderness localized on direct pressure with point of a single finger is evident, abdominal resistance is noticeable, adhesions occur as the peritonitis extends, agglutinating contiguous surfaces and happily hedging in the commencing suppuration and abscess; septic fever, tympanites, and increased pulse follow as a rule, varying with the intensity and rapidity in the spread of the inflammation.

*General gangrene* of the appendix is the most formidable of all these lesions, and death may ensue within a few hours, such is the suddenness of the onslaught and the rapidity of the gangrene and even before rupture, such is the widespread peritonitis produced and consequent shock and collapse.

In *local gangrene* and *ulceration* the general peritonitis may be prevented by fortunate early adhesions, but the perforation is usually sudden enough to permit the escape of gaseous or other contents, with rapidly developing peritonitis.

When foreign particles find lodgment in the appendix they may produce a mild form of inflammation, recurring with varying frequency (recurrent appendicitis) causing adhesions, the attacks gradually increasing in severity until either symptoms of obstruction are present or abscess and general peritonitis ensue.

In *gangrene* of the appendix the symptoms do not differ materially from those in perforation of this organ. The following may be taken as a typical case. It occurred in the practice of Drs. Walker & McBurney, of New York.* "A man, fifty-six years old, heavy build and in excellent health, was seized quite suddenly at 9 P. M. with nausea, vomiting and general abdominal pain. His temperature remained at 100° until evening of next day. Forty-eight hours after the onset of the attack the abdomen was moderately distended, rigidity of right abdominal muscles was highly developed, and great tenderness on pressure was noted at the ileo-cæcal upper region. He had the gray tired look of commencing sepsis. Within two hours the temperature had risen from 100° to 102.5°, the pulse was full and bounding. At midnight, fifty-one hours after the attack began, the operation was performed. The appendix, curved like the letter S, five inches long, as large as a man's thumb, extremely dis-

*Medical Record, April 6, 1892, p. 424.

tended with soft fæces and completely gangrenous, was found lying to the outside of the ascending colon well in the loin. It was not perforated and there was no pus. The usual toilet and dressing was done and the patient recovered.

*Subacute* and *chronic*, or *recurrent appendicitis*. The following case will, I think, suffice in the study of this group.

In April, 1890, a young man about twenty years old came to me from Dr. Ground, of New Hampshire. He had had twelve attacks of appendicitis in eighteen months. They had varied in intensity, were generally not severe. There was always pain in the right iliac region, abdominal tension (often mistaken for tumefaction), and occasionally a temperature as high as 101° F. He was incapacitated for work from one to two weeks during each attack. While preparing him for operation an attack came on with the usual symytoms of other seizures. It subsided in ten days, and I then removed the appendix which was adherent to the iliac fascia. The peritoneum was thickened and there were numerous adhesions about the cæcum. The appendix was not more than one inch in length, and about three-eighths of an inch in diameter, was empty and not inflamed or seemingly diseased. The patient recovered promptly and has not had an attack since that date, notwithstanding the character of his work, which is that of a porter at a hotel.

When appendicitis occurs in which the inflammation extends gradually, yet steadily through the wall of this organ, involving the peritoneum, adhesions rapidly occur, and suppuration being established, the pus is circumscribed or walled off from the general peritoneal cavity and produces the usual symptoms of abscess, along with localized or general peritonitis. In this variety there are not present the symptoms of shock which characterize gangrene, perforation or rupture of a peri-appendicular abscess. Though less dangerous, these cases require unremitting vigilance. It is true that in many instances resolution occurs, even after well-marked tumefaction is present, with fairly high temperatures, tympanites and local peritonitis, but I believe in these cases the tumefaction is chiefly due to localized muscular tension and to limited peritonitis with adhesions rather than to abscess.

In these cases of gradually developing peri appendicular abscess the surgeon should be prepared for operation in the first few days of the attack. The sharply defined limit of tenderness, increasing swelling and muscular resistance, persistent high temperatures (101° to 102.5° F.) and tympanites, all point to increased formation of pus and strongly suggest operative interference. If all such cases were thus early operated upon, the general mortality after peri-appendicular abscess would, in my opinion, be greatly decreased.

Within the last few months I have seen three such cases perish from rupture of the abscess between the seventh and tenth days from the first symptoms of the attack, and in each of these the conditions were very favorable for operation after the abscess was well advanced. In one, operation was advised on the fifth day, but declined. The patient died from peritonitis and collapse on the tenth day. An enormous abscess filled the right ileo-cæcal region and had ruptured. In the second case, on the night of the eighth day the patient "felt something give way;" collapse ensued, and as he was moribund I declined to operate. He died in two hours.

In the third case, on opening the abdomen fetid pus was found in the general peritoneal cavity, and although a careful toilet was made he died in five hours. His condition was considered desperate and almost hopeless at the time of operation, which had been too long deferred.

In cases of suspected abscess I do not think we should use an aspirating needle in exploration. It may be followed by serious consequences. When the symptoms point to pus accumulation, the most surgical procedure is to cut down upon the swelling. If adhesions have occurred and you get directly into the abscess, the treatment is the drainage tube and irrigation with Thiersch's solution. If the general peritoneal cavity is first entered, the abscess can be readily recognized and located by enlarging the incision in the abdominal wall, if necessary. Rather than risk the escape of pus into the peritoneal cavity a posterior opening should be made, entering the abscess through formed adhesions and draining out from behind. The anterior incision facilitates the entrance to the abscess from behind. The anterior wound should be closed throughout. If only a very small pus sac is found it may be opened, its contents sponged out, the cavity scraped and cleansed with sponges, rinsed with 1-1000 bichloride solution, and the wound packed with iodoform gauze. No posterior incision is indicated here.

When an abscess has burst into the general cavity a thorough and careful toilet should be performed, everything sponged out dry, especial attention given to the pelvic cavity. Iodoform packing is demanded.

*Operative technique in the removal of the appendix.* An incision about five inches long is made through the abdominal wall parallel with the linea alba. The center of this incision should be about one-half of an inch below a line drawn from the anterior superior spine of the ilium to the umbilicus. All bleeding should be stopped by ligature before the peritoneum is opened. When this is done any loops of small intestine which may protrude should be gently pushed toward the median line and held away by sterilized gauze napkins. The cæcum is readily seen, and may be recognized by the longitudinal band, which, if followed downward, leads directly to the appendix. Should no adhesions be found, the end of the cæcum may be slightly lifted and thus bring the appendix into view, when it can be dissected from its peritoneal attachments, commencing at the apex. About one-fourth of an inch from its junction with the large intestine, a strong silk ligature is applied around it, and the appendix cut away with the scissors from one-eighth to one-fourth of an inch beyond the ligature. The end of the stump is rendered aseptic by thoroughly applying over its cut surface a solid tablet of mercuric bichloride. The ends of the ligature are cut off close to the knot after the toilet of the peritoneum. A piece of rubber tissue protective, disinfected by immersion in 1-5000 cool bichloride solution is now placed so as to wall off the small intestines from the stump, and against this tissue is placed the iodoform gauze, which fills the wound and projects to the surface through its lower one or two inches, which is not closed by sutures. The rubber tissue is used because it does not adhere to the intestines as does the gauze. The upper remaining portion of the wound is closed as after an ordinary laparotomy. This dressing may remain for from three to ten days, preferably about seven days, when the gauze and tissue protective are pulled out, the wound irrigated with 1-3000 bichloride solution. A small strip of iodoform gauze or a soft rubber tube may be carried down to the bottom of the wound to insure drainage, and this finally removed in another week or ten days. For fear of ventral hernia the patient should keep in bed for four or six weeks, to permit the lower part of the wound to close by strong cicatricial adhesion.

When adhesions between contiguous loops of intestines and the appendix or cæcum are met with these should be carefully separated, and upon the discovery of pus or extravasated matter this should be mopped out with sponges, taking care to prevent, if possible, the contact of septic matter with the general peritoneal cavity. The use of sterilized gauze napkins may aid in walling off the general cavity.

When the appendix is gangrenous or greatly distended, or has already been perforated, very considerable care is essential in dissecting it loose, either to prevent rupture or to avoid the escape of its contents.

When a large agglutination is encountered and the presence of a large quantity of collected pus is evident, it is safer to use the anterior incision which led to this discovery as a guide to the entrance of the abscess from a posterior and retro-peritoneal incision through which drainage can be effected. The anterior wound can then be closed throughout by sutures; if not contra-indicated I prefer good sterilized silk for sutures, passing through the skin and carefully going through each layer of fascia and muscles, and the abdominal peritoneum at least one-fourth of an inch from the cut edge of the peritoneum.

When the general toilet of the peritoneum is indicated I prefer irrigating this cavity with sterilized water (just boiled and allowed to cool down to about 100 degrees F.) This is carried in from an elevated irrigator through a stiff, blunt gutta-percha tube (boiled before using). As the general peritoneal cavity becomes filled the fluid flows out through the opened wound. The remainder is sponged until the cavity is dry and clean. I have once made a second opening in the median line in order to drain the deep pelvis. This is rarely indicated.

In conclusion it may be said that, contrary to former teaching, so-called perityphlitis and perityphlitic abscesses are intra-peritoneal lesions. They seemed to be retro-peritoneal after adhesions had taken place.—*The International Journal of Surgery.*

